# *Tulasnella calospora* (UAMH 9824) retains its effectiveness at facilitating orchid symbiotic germination in vitro after two decades of subculturing

**DOI:** 10.1186/s40529-021-00321-w

**Published:** 2021-09-27

**Authors:** Lawrence W. Zettler, Caleb J. Dvorak

**Affiliations:** 1 Orchid Recovery Program, Department of Biology, Illinois College, 1101 W College Avenue, Jacksonville, IL 62650 USA; 2 Micropropagation Coordinator, Missouri Botanical Garden, 4344 Shaw Blvd., St. Louis, MO 63110 USA

**Keywords:** Conservation, Habitat preservation, Mycorrhizal fungi, Symbiotic, *Rhizoctonia*

## Abstract

**Background:**

The technique of symbiotic germination—using mycorrhizal fungi to propagate orchids from seed in vitro—has been used as one method to cultivate orchids in North America and abroad for > 30 years. A long-held assumption is that mycorrhizal fungi used for this purpose lose their effectiveness at germinating seeds over time with repeated subculturing.

**Results:**

We provide evidence for the lingering efficacy of one particular strain of *Tulasnella calospora* (266; UAMH 9824) to stimulate seed germination exemplified by the North American terrestrial orchid, *Spiranthes cernua*, as a case study. This fungus was originally acquired from roots from *Spiranthes brevilabris* in 1999 and sub-cultured during the two decades since. Seeds inoculated with the fungus in vitro developed to an advanced protocorm stage after 16 days, and leaf elongation was pronounced after 42 days. In a pilot study, seedlings co-cultured with *Tulasnella calospora* 266 were deflasked after 331 days and later transferred to soil under greenhouse conditions where they eventually initiated anthesis. During the course of two decades, seeds of 39 orchid species, cultivars and hybrids spanning 21 genera, germinated in vitro co-cultured with *Tulasnella calospora* 266. These orchids included temperate terrestrials and tropical epiphytes alike.

**Conclusions:**

The sustained effectiveness of this fungus is noteworthy because it argues against the concept of mycorrhizal fungi losing their symbiotic capability through prolonged subculturing. This study serves as an example of why in situ habitat preservation is essential for the conservation of orchids as a source of potentially useful mycorrhizal fungi.

## Background

**Fig. 1:**
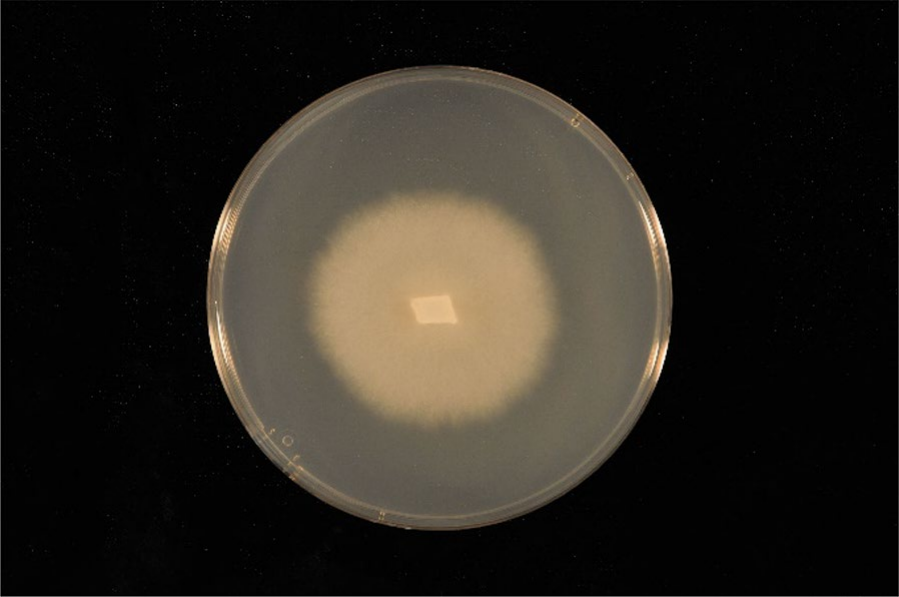
Pure culture of *Tulasnella calospora* 266 (UAMH 9824) shown within a 9 cm diam. petri dish containing Potato Dextrose Agar > 10 days after inoculation. The culture is recognizable by the smooth, cream-colored mycelium and smooth colony margins. Photo courtesy of Michael E. Kane

The technique of symbiotic germination has been used as one method to propagate orchids from seed for over 30 years (Clements et al. 1986). In 1999, an attempt was made to isolate fungi for this purpose to propagate *Spiranthes brevilabris* from seed in vitro—a rare species restricted to a single population consisting of 152 individuals clustered within a small (21,600 ha) state park in Florida, USA (Stewart et al. 2003). One fungus was subsequently isolated and provisionally identified as a member of the *Rhizoctonia* complex, the group of higher fungi (basidiomycetes) known for forming mycorrhizal associations with photosynthetic orchids worldwide. This fungus matched published descriptions for *Epulorhiza repens* (N. Bernard) R. T. Moore (1987), a common ubiquitous species. One year after its isolation, seeds collected from the donor population were inoculated with the fungus in vitro using standard protocols (Dixon 1987), and seed germination and seedling development rapidly ensued. Only 133 days after sowing, > 165 laboratory-grown seedlings were reintroduced to Florida, and 100% survived after one month, effectively doubling the size of the orchid population. Six months later, 17 of the reintroduced orchids initiated anthesis (Stewart et al. 2003). This fungus was then permanently retained and catalogued at Illinois College as Isolate 266 (Fig. 1) where it was then placed in cool (4 °C) storage for future use. In addition, the fungus was also deposited into the University of Alberta Microfungus Herbarium in Canada as UAMH 9824 for safekeeping under cryopreservation.

In the decades that followed, this fungal isolate was used in other symbiotic germination experiments involving a wide range of additional orchid species. Among the taxa successfully propagated to the leaf-bearing stage included epiphytes (*Cyrtopodium punctatum* and *Epidendrum nocturnum*) as well as terrestrials (e.g., *Habenaria repens* and *Platanthera holochila*) including five additional *Spiranthes* species (Massey and Zettler 2007). The use of UAMH 9824 in germination experiments extended into a second decade where it retained its effectiveness at facilitating advanced seedling development, earning it the nickname as the super-fungus. At the same time, modern molecular techniques, namely Sanger sequencing of barcode regions like Internal transcribed Spacer of rDNA (ITS), were being perfected that enhanced fungal identification within the *Rhizoctonia* complex to which members of the genus *Epulorhiza* belonged. These new techniques also coincided with the abandonment of the anamorphic classification system in higher fungi in favor of the teleomorphic binomial. Hence, members of the genus *Epulorhiza* R. T. Moore are now placed in the genus *Tulasnella* J. Schröt, and *E. repens* is now regarded as *Tulasnella calospora* (Bourdier) Juel, which we refer to herein as *Tulasnella calospora* 266.

The ability of *Tulasnella calospora* 266 to retain its efficacy during the span of two decades counters a long-held assumption that fungal endophytes lose their ability to stimulate seed germination through repeated subculturing (Bernard 1909; Alexander and Hadley 1983). At the time of this writing (2021), this fungus has facilitated in vitro symbiotic germination of two epiphytic orchids native to Ecuador (Quijia-Lamiña 2020) and is also now being used by at least one hobbyist to propagate several terrestrial orchids including one native to Europe (*Dactylorhiza praetermissa*; D. Martin, pers. com.). In this paper, we provide evidence for the sustained effectiveness of *Tulasnella calospora* 266 to stimulate seed germination spanning two decades, using *Spiranthes cernua* as a case study. The effectiveness of this fungus is compared to three other fungal isolates as well as two types of asymbiotic media. We conclude by presenting a comprehensive list of the orchid species propagated with this fungus during the past 20 years to the best of our knowledge.

## Methods

### Maintenance of *Tulasnella calospora* 266

Two types of agar media were employed in the care and maintenance of *Tulasnella calospora* 266 over the course of two decades. For short-term maintenance at ambient temperature, potato dextrose agar (PDA) was used (Difco™ #213400, Difco Laboratories, Detroit, MI, USA). Cultures were maintained on PDA in 9 cm diam. petri dishes that were wrapped in Parafilm® “M” (Menasha, WI, USA) to seal in moisture. At 3–6 month intervals, a 1 cm^3^ block of fungal inoculum from the outer margin of the colony was subcultured to a new PDA plate using a sterile scalpel, and the process was repeated. In cases when the fungus failed to initiate growth usually > 6 months, a back-up culture of the fungus was restarted on PDA. Back-up cultures consisted of maintaining *T. calospora* 266 in refrigeration (4 °C) on oatmeal agar (OMA) slants in screw-cap test tubes for 1–2 years. OMA consisted of 2.5 g/L Quaker Oats®, Chicago, IL, USA; 7.5 g/L Bacto™ Agar (#214010), Becton, Dickinson and Co., Sparks, MD USA.

### Seeds, mycorrhizal fungi, and agar media

Seeds of *Spiranthes cernua* (L.) Rich were collected by E. Esselman on 25 September 2018 from a natural population located in Bond Co., Illinois that contained ca. 50 individual orchids. Multiple mature capsules derived from natural pollination among 10 different individuals were removed from inflorescences just prior to dehiscence, cleaned of debris and immediately dried over CaSO_4_ (Drierite, W.A. Hammond Co., Xenia, Ohio, USA) at ambient temperature (22 °C) for 10 days. Seeds were then removed from capsules, pooled into a single airtight vial, and stored at 4 °C for 61 days in darkness until use.

Four fungal isolates were tested for their ability to facilitate seed germination of *S. cernua*. Two were from Florida and assignable to the genus *Tulasnella* including 266, and the other two consisted of *Ceratobasidium* strains from prairie habitats similar to where *S. cernua* grew naturally (Table 1). Two types of asymbiotic media were chosen for comparative purposes: P723 Orchid Seed Sowing Medium, and B141 Terrestrial Orchid Medium (*Phyto*Technology Laboratories®, Shawnee Mission, KS, USA). The media chosen for symbiotic germination consisted of standard oatmeal agar or OMA (mentioned previously). All media were prepared using RO water with the pH adjusted to 5.6–5.8 prior to autoclaving using NaOH or HCl.

**Table 1:** The sources for the four strains of mycorrhizal fungi used in symbiotic germination of *Spiranthes cernua*

Fungal Isolate ID #	Provisional Identity	Source	Year Isolated	Reference
266 (UAMH 9824)	*Tulasnella calospora*	*Spiranthes brevilabris* Goethe State Forest Levy Co., FL	1999	Stewart et al. (2003)
427 (UAMH 11955)	*Tulasnella* sp.	*Dendrophylax porrectus* Florida Panther NWR Collier Co., FL	2016	L. Zettler (unpub, data.)
PP4	*Ceratobasidium* sp.	*Platanthera peramoena* Fayette Co., IL	2018	E. Esselman (unpub. data)
EE465	*Ceratobasidium* sp.	*Platanthera leucophaea* Jackson Co., IA	2018	E. Esselman (unpub. data)

### Seed sowing, inoculation, and incubation in vitro

Seeds were surface-sterilized within packets prepared from AeroPress coffee filters (AeroPress®, Palo Alto, CA, USA). Seeds were placed in the center of each coffee filter, which was folded around the seeds and stapled shut into a packet. The number of seeds delivered into each packet was carried out volumetrically with a micro spatula. The volume was estimated at 4 mm^3^ based on a small strip of seeds on the end of the spatula measuring ca. 4 mm long, 1 mm deep, and 1 mm high. Considerable care was given to adding an equal number of seeds to each packet. A total of 70 packets were constructed. The packets were pre-soaked by immersing in reverse osmosis (RO) water with 1 drop of Tween® 20 per 100 mL, and agitated on an orbital shaker at 130 rpm for 10 min. Packets were then decontaminated by immersing them in an RO water solution containing 0.5% NaDCC + 1 drop Tween® 20 per 100 mL that was agitated on the shaker at 130 rpm for 30 min. Packets were removed from the solution under a laminar flow hood and transferred to a container of sterile water to remove traces of the decontamination solution for ca. 5 min. Packets were then removed from the sterile water and cut open with a scalpel under a laminar flow hood and seeds from each packet were spread onto the agar surface within 9 cm diam. petri dishes (25 mL agar/dish). Because some seeds lacked embryos, only those that contained embryos were counted and included in our data set. This yielded a range of 37–125 seeds per dish. With the exception of controls, all oatmeal agar plates were inoculated with a given fungus by adding a 1 cm^3^ block of inoculum to the center of each petri plate. Seven treatments were tested: OMA control, OMA + *Tulasnella calospora* 266, OMA + *Tulasnella* 427, OMA + *Ceratobasidium* PP4, OMA + *Ceratobasidium* EE465, *Phyto*Tech P723, and *Phyto*Tech B141.

**Fig. 2:**
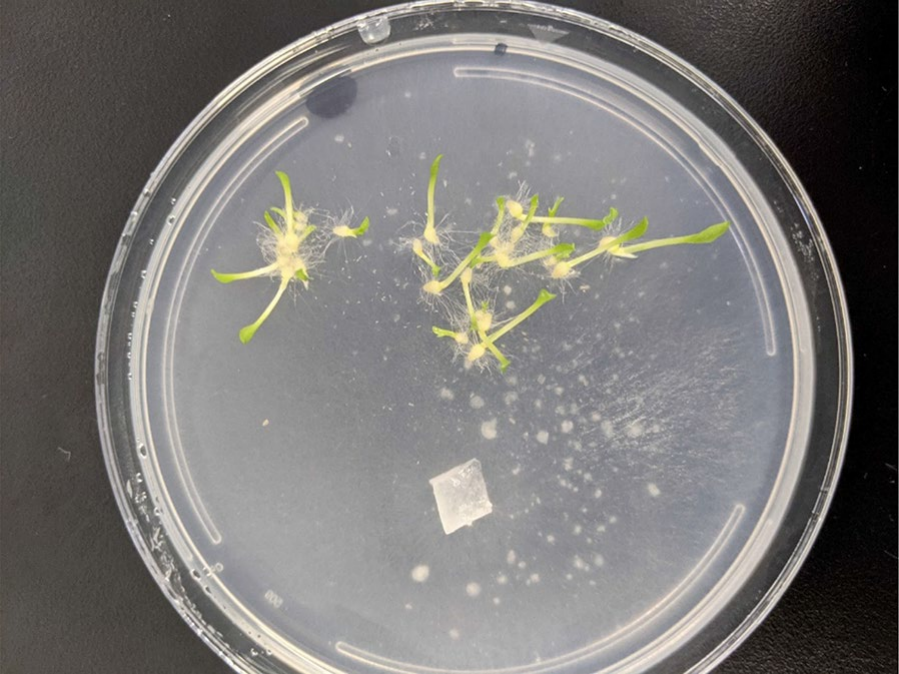
Leaf-bearing (Stage 5) *Spiranthes cernua* seedlings on the surface of oatmeal agar (OMA) co-cultured with *Tulasnella calospora* 266. The photo was taken 42 days after sowing and incubation in darkness at ambient temperature followed by 7 days under a white light photoperiod

Following sowing/inoculation, plates were sealed in polyethylene film, stacked into a Styrofoam box with a lid, and incubated in darkness within a growth chamber at 25 °C. After 16 days, plates were inspected visually for contamination and early signs of germination, and promptly returned to the growth chamber. After 42 days (post-sowing), all plates were removed from growth chambers for data collection and observation. Seed germination and seedling development were scored on a scale of 0–5 in accordance with Zettler et al. (1995) where: Stage 0 = no germination; 1 = rupture of testa by enlarged embryo (= germination); 2 = rhizoid formation; 3 = elongation of protocorm (shoot formation); 4 = appearance of first leaf within shoot region; and 5 = elongation of first leaf. Data were analyzed using general linear model procedures multivariate analysis of variance (MANOVA) and mean separation at α = 0.05 by IBM SPSS Statistics for Windows, Version 26.0 for Windows subprogram (Armonk, NY: IBM Corp.). Plates that harbored leaf-bearing seedlings were transferred to a second growth chamber and illuminated under an 18 h photoperiod (L:D 18:6 h) to induce photosynthesis in the shoot region (Fig. 2). Illumination was provided by full spectrum bulbs (32 Watt T8 fluorescent, Philips™ fluorescent tube model F32T8/TL741 Alto II), and irradiance was measured to be 40 µmolm^−2^ s^−1^ at the plate surface. Temperatures during the light and dark cycle were adjusted to 25 and 23 °C, respectively.

### Seedling establishment ex vitro

In a pilot study that led to the in vitro techniques described above, seedlings with sizable leaves were removed from petri dishes and transferred to taller 8 oz. (ca. 250 mL) polypropylene vessels containing 80 ml OMA, 134 days after sowing. These seedlings, which were obtained using two of the fungal isolates (*Tulasnella calospora* 266 and *Ceratobasidium* PP4), were placed onto the surface of OMA in the vessel instead of a mineral-enriched salt medium. This decision was based on concerns that higher levels of mineral salts such as nitrogen may have a negative impact on plant-fungal symbiotic relationships. Each vessel contained 12 seedlings (seedlings from different treatments were not combined into the same vessel). Vessels were placed back into the growth chamber for further growth. After 331 days post-sowing, seedlings were removed from their vessels and the media was rinsed from the roots. They were placed into sealed plastic bags containing damp sphagnum (peat) moss and vernalized in a refrigerator at 4 ℃ for 3 months. After vernalization, seedlings were planted in potting mix consisting of aged pine bark, Canadian sphagnum moss, and perlite (Ball Horticultural Co, West Chicago, IL, USA) and placed in a greenhouse to continue growing leading to anthesis.

## Results

**Fig. 3:**
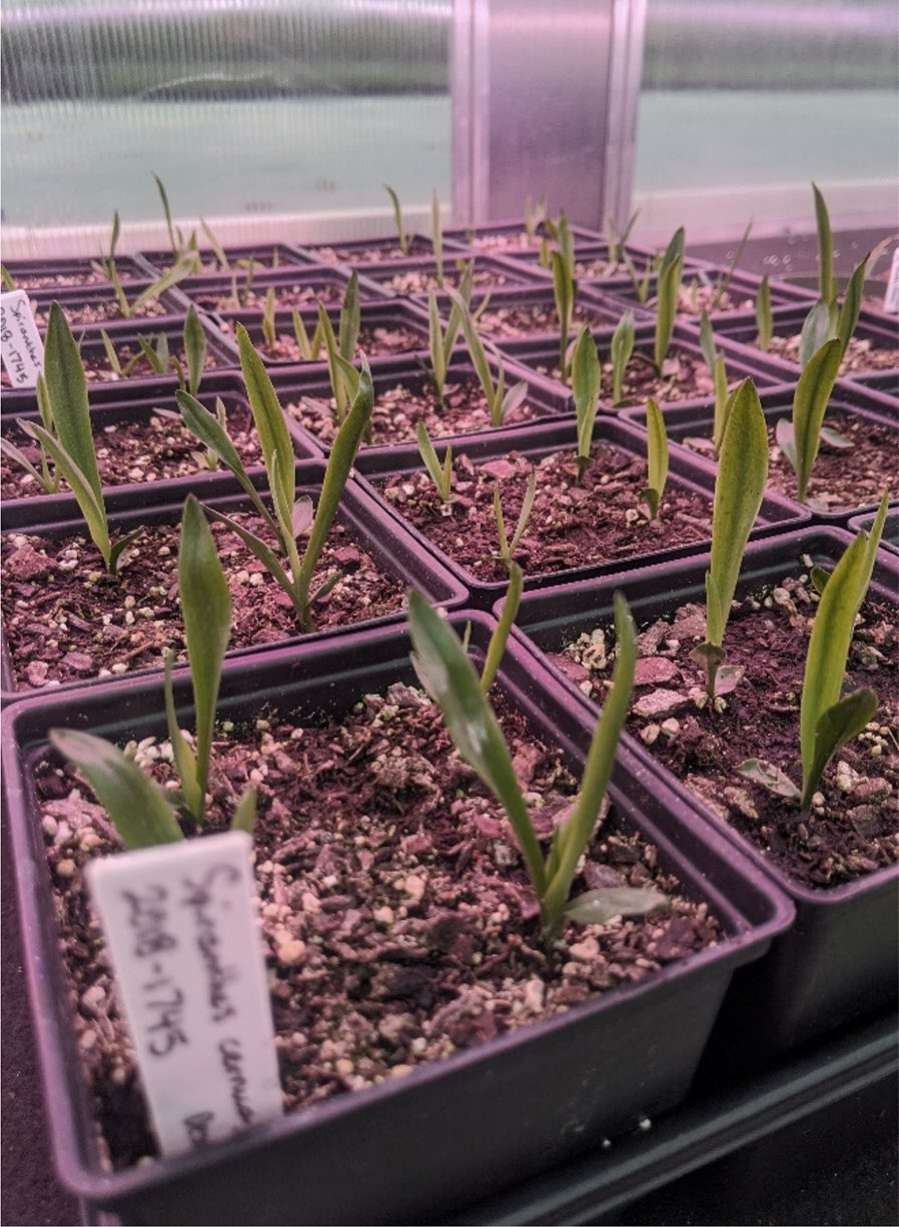
Greenhouse-established seedlings of *Spiranthes cernua* on soil after being vernalized at 4 °C prior for three months in darkness. These seedlings initiated anthesis after 421 days (331 days in lab culture plus 3 months in vernalization)

Seed germination was observed in all seven treatments 16 days after sowing followed by incubation in darkness. Of the seven treatments, seeds inoculated with *Tulasnella calospora* 266 progressed to Stage 4—the highest growth stage amongst the treatments at that point in time. After 42 days of incubation, seeds inoculated with *Tulasnella calospora* 266 and *Ceratobasidium* PP4 resulted in the highest percent germination observed (33.7 and 34.6%, respectively) followed by *Tulasnella* 427 (29.6%). The MANOVA revealed germination across treatments was significant, F (6,62) = 72.65, *p* < 0.01 (Table 2). After 42 days of incubation, 5.8% of seedlings co-cultured with *Tulasnella calospora* 266 developed to Stage 5 (leaf elongation) compared to only 1.2% of seedlings in the *Ceratobasidium* PP4 treatment (Fig. 3). None of the other five treatments, including OMA + *Tulasnella* 427 developed beyond Stage 2 at the 42 day mark (Table 2).

**Table 2:** A comparison of in vitro symbiotic vs. asymbiotic seed germination of *Spiranthes cernua* by growth stage 42 days after sowing

Treatment	*n*^a^	# Seeds	Stage 0	Stage 1	Stage 2	Stage 3	Stage 4	Stage 5^b^	% Germination (± SE)^c^
P723	10	730	700	25	5	0	0	0	3.68 (1.16)^a^
B141	9	634	584	39	11	0	0	0	8.94 (2.47)^b^
OMA + 266	10	690	466	113	10	29	32	40 (5.8)	33.73 (2.73)^c^
OMA + P44	10	735	481	89	51	55	50	9 (1.2)	34.65 (1.84)^c^
OMA + EE465	10	745	672	65	8	0	0	0	9.65 (0.91)^b^
OMA + 427	10	848	598	242	8	0	0	0	29.56 (1.68)^c^
OMA control	10	665	663	2	0	0	0	0	0.31 (0.31)^a^

Note that *Tulasnella calospora* 266 facilitated the largest percentage of seedlings (> 5.8%) to the highest growth stage for all treatments including the three symbiotic treatments that utilized fungi isolated more recently (2016 and 2018)

^a^Number of replicate petri plates (*n*) for a given treatment. Unequal sample sizes resulted when contaminated plates were discarded

^b^Values in parentheses reflect the percentage of seeds in a given treatment that developed to Stage 5

^c^Means denoted by the same letter were not significantly different (α = 0.05)

In the pilot study, deflasked seedlings from the OMA + *Tulasnella calospora* 266 and OMA + *Ceratobasidium* PP4 treatments continued development leading to soil transfer under greenhouse conditions after 421 days (331 days in lab culture plus 3 months in vernalization; Fig. 3). Seeds sown on the asymbiotic media (B141 and P723) failed to progress beyond Stage 4 after 421 days.

## Discussion

**Fig. 4:**
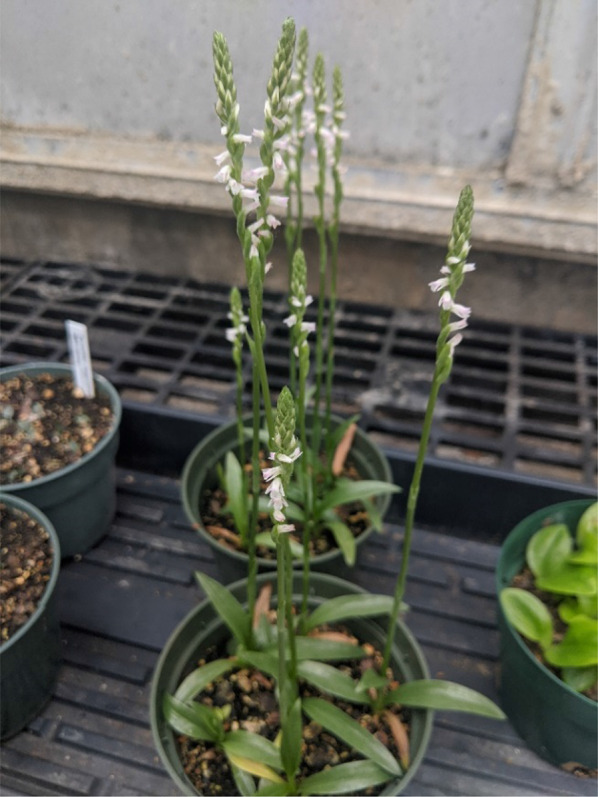
*Spiranthes sinensis* cultivated to the blooming stage at the Missouri Botanical Garden (MBOT) after seeds were symbiotically cultured with *Tulasnella calospora* 266 in vitro following the same protocols for *S. cernua*

**Fig. 5:**
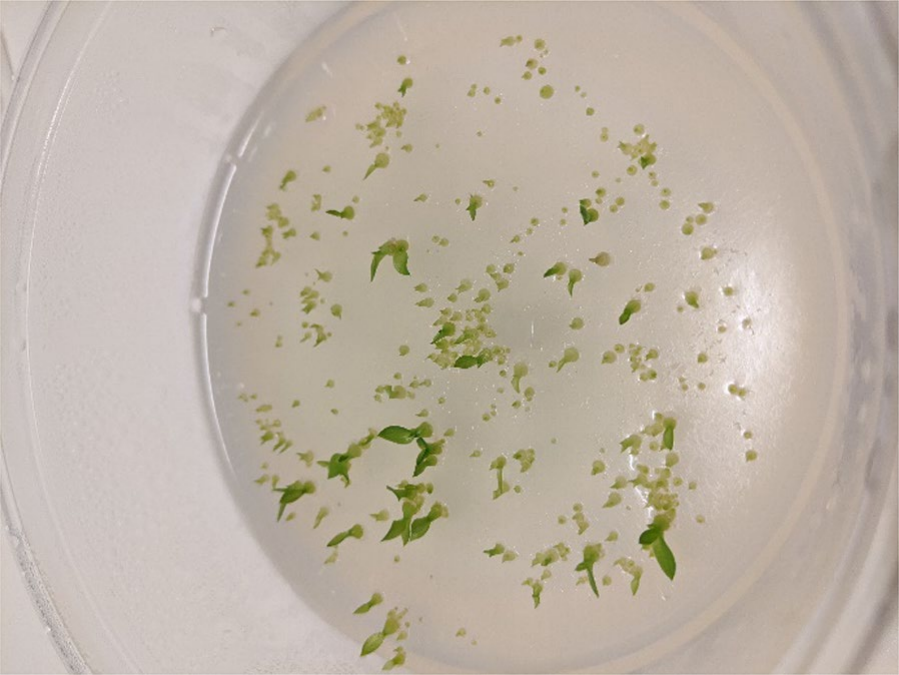
Seedlings of a *Cattleya* hybrid in various stages of development in symbiotic in vitro culture with *Tulasnella calospora* 266 in an 8 oz. polypropylene vessel. Stage 5 seedlings are those with the largest leaves, mostly shown at the left of the image

**Fig. 6:**
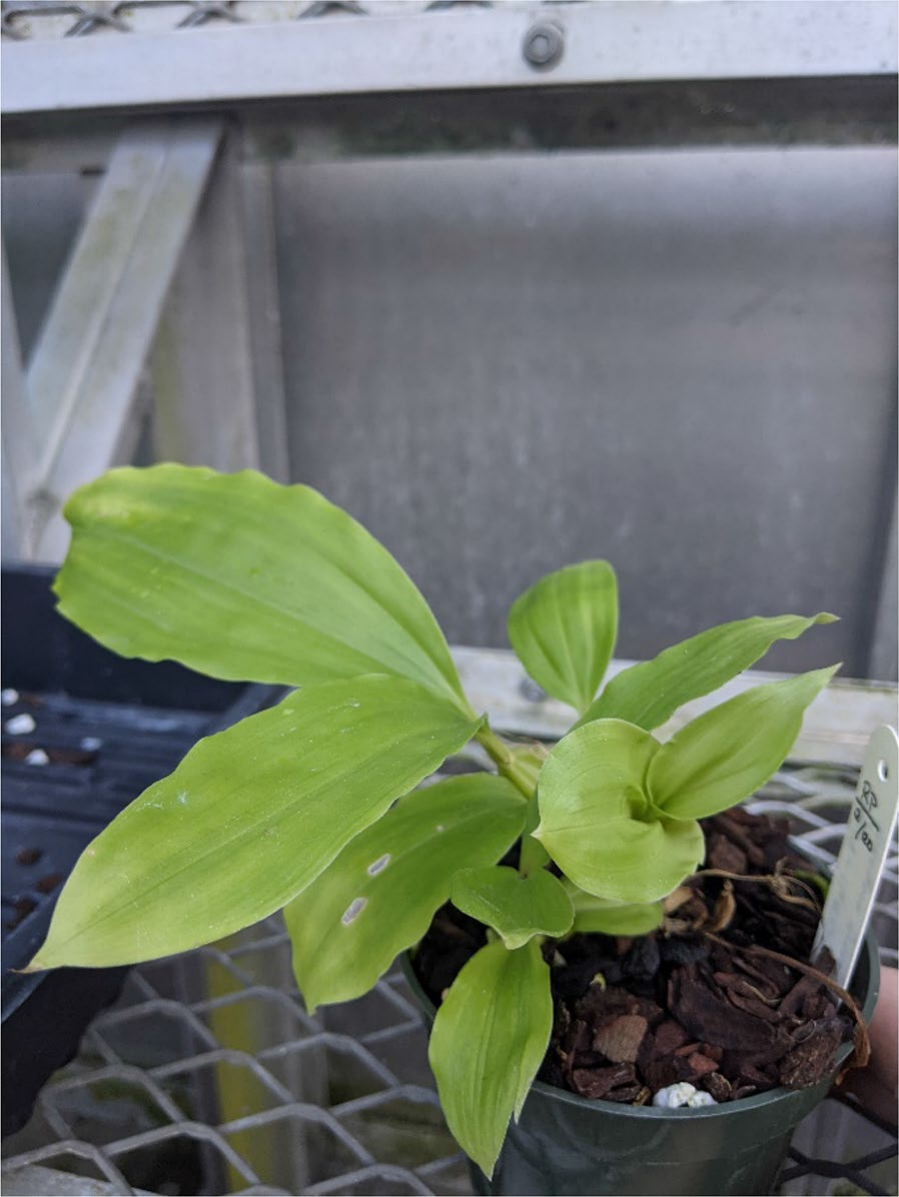
Greenhouse established seedling of the South American native, *Gongora grossa*, that was cultivated at the Missouri Botanical Garden in vitro using *Tulasnella calospora* 266. Approximately 17% of the seeds germinated using the fungus

This case study demonstrated the effectiveness of *Tulasnella calospora* 266 (UAMH 9824) at facilitating seed germination and seedling development of *Spiranthes cernua* despite repeated subculturing over the course of 20 years. This efficacy is evidenced by the rapid (42 day) development of seedlings to Stage 5 in symbiotic culture (Table 2) compared to two other fungal isolates acquired more recently (2016 and 2018). This study is just one example of the many different types of orchids that have been propagated to date spanning North America and abroad, even to the blooming stage such as *Spiranthes sinensis* (Fig. 4; Table 3). The sustained effectiveness of *Tulasnella calospora* 266 to propagate a broad range of species, commercial hybrids (*e.g*., *Cattleya*; Fig. 5) and species under cultivation (e.g., *Gongora grossa*; Fig. 6) is noteworthy because it argues against the concept of mycorrhizal fungi losing their symbiotic capability through prolonged subculturing (Bernard 1909; Alexander and Hadley 1983). Consequently, this study may have merit for improving conservation and horticulture alike. The use of this fungus to germinate seeds of a North American hybrid (*Platanthera integrilabia* x *ciliaris*) by at least one hobbyist (D. Martin) suggests that *Tulasnella calospora* 266 could also be utilized in developing new varieties for horticulture, as well as answer fundamental ecological questions.

**Table 3:** A comprehensive list of the orchids propagated from seed in vitro using *Tulasnella calospora* 266 during the past two decades

Orchid species	Seed origin	Highest growth stage	References and notes
*Anacamptis morio*	England	Stage 2 (in progress)	D. Martin (unpub. data)
*Barkeria spectabilis* x *skinneri* Aphrodite	MBOT	Stage 5	C. Dvorak (unpub. data)
*Bletia striata*	MBOT	Blooming size	C. Dvorak (unpub. data)
*Bletilla ochracea*	cultivated	Stage 1 (in progress)	D. Martin (unpub. data)
*Brassavola nodosa*	MBOT	Stage 3	C. Dvorak (unpub. data)
*Calopogon* Fluffy (*tuberosus* x *multiflorus*)	cultivated	Stage 3 (in progress)	D. Martin (unpub. data)
*Calopogon tuberosus*	MBOT	Stage 5	C. Dvorak (unpub. data)
*Cattleya* hybrid	MBOT	Stage 5	C. Dvorak (unpub. data)
*Cyrtopodium punctatum*	Florida	Stage 5 (deflasked)	S. Stewart (unpub. data)
*Dactylorhiza incarnata*	UK	Stage 2 (in progress)	D. Martin (unpub. data)
*Dactylorhiza praetermissa*	Netherlands	Stage 3 (in progress)	D. Martin (unpub. data)
*Dactylorhiza purpurella*	UK	Stage 2 (in progress)	D. Martin (unpub. data)
*Dactylorhiza romona*	UK	Stage 2 (in progress)	D. Martin (unpub. data)
*Encyclia tampensis*	MBOT	Stage 5 (deflasked)	C. Dvorak (unpub. data)
*Epidendrum geminiflorum*	Ecuador, SA	Stage 5	Quijia-Limiña (2020)
*Epidendrum nocturnum*	Florida	Seedlings reintroduced	Zettler et al. (2007)
*Gongora grossa*	MBOT	Stage 5 (deflasked)	C. Dvorak (unpub. data)
*Goodyera pubescens*	MBOT	Stage 2	C. Dvorak (unpub. data)
*Gymnadenia conopsea*	UK	Stage 1 (in progress)	D. Martin (unpub. data)
*Habenaria macroceratitis*	Florida	Stage 3	Stewart and Zettler (2002)
*Habenaria odontopetala*	Florida	Stage 4	S. Stewart (unpubl. data)
*Habenaria repens*	Florida	Blooming size	Stewart and Zettler (2002)
*Liparis hawaiensis*	Hawaii	Stage 2 (in progress)	C. Fryrear and A. Zettler (unpub. data)
*Piperia unaluscensis*	Washington	Stage 4	S. Stewart (unpub. data)
*Platanthera bifolia*	UK	Stage 2 (in progress)	D. Martin (unpub. data)
*Platanthera blephariglottis*	Pennsylvania	Stage 2 (in progress)	D. Martin (unpub. data)
*Platanthera ciliaris*	Cultivated Indiana SE USA	Stage 3 (in progress) Stage 4 (in progress) Stage 4	D. Martin (unpub. data) S. Renken (unpub. data) Hartsock et al. (2003);
*Platanthera dilitata*	California	Stage 3 (in progress)	D. Martin (unpub. data)
*Platanthera integrilabia* x *ciliaris*	Cultivation	Stage 3 (in progress)	D. Martin (unpub. data)
*Platanthera (Peristylus)* *holochila*	Hawaii	Stage 4	Zettler et al. (2007)
*Polystachya concreta*	Ecuador, SA	Stage 5	Quijia-Limiña (2020)
*Prosthechea cochleata* var *triandra*	Florida	Stage 2	S. Stewart (unpub. data)
*Spathoglottis plicata*	Florida	Blooming size	S. Stewart (unpub. data)
*Spiranthes brevilabris*	Florida	Blooming size	Stewart et al. (2003); Stewart and Kane (2007)
*Spiranthes cernua*	Illinois	Blooming size	current study
*Spiranthes cernua*	Florida	Blooming size	S. Stewart (unpub. data)
*Spiranthes delitescens*	NA	Stage 4	A. Hicks (unpub. data)
*Spiranthes longilabris*	Florida	Stage 4	L. Zettler and K. Piskin (unpub. data)
*Spiranthes magnicamporum*	Missouri	Stage 4	C. Dvorak (unpub. data); Wagoner et al. (2002)
*Spiranthes odorata*	Florida	Stage 4	S. Stewart (unpub. data)
*Spiranthes sinensis*	MBOT	Blooming size	C. Dvorak (unpub. data)
*Spiranthes triloba*	Florida	Blooming size	S. Stewart (unpub. data)
*Spiranthes vernalis*	Illinois	Stage 5	C. Dvorak (unpub. data)

Growth stages noted as in progress are symbiotic germination trials that are currently underway. Seed origin listed as MBOT were from collections made by the Missouri Botanical Garden under permit

In the pilot study, we questioned whether the transfer of symbiotically-grown *S. cernua* seedlings on OMA to a more mineral salt-enriched medium might lead to mineral toxicity, and for this reason we chose to use the same OMA medium instead. This resulted in seedlings that appeared to display symptoms of nitrogen deficiency evidenced by chlorosis in the apical leaves and loss of most basal leaves closest to the crown. The subsequent transfer of these seedlings to potting mix after vernalization resulted in rapid growth suggesting that the minerals in the mix provided an adequate supply of nutrients (*e.g*., nitrogen). In follow-up experiments (ongoing), seedlings on OMA transferred directly to pasteurized potting mix instead of OMA showed no signs of nitrogen deficiency (C. Dvorak, pers. obs.). Consequently, we recommend that other researchers consider transferring seedlings on OMA directly to pasteurized potting soil, and that the use of media containing mineral-salts may not be necessary.

Another highlight of this study concerns the origin of *Tulasnella calospora* 266, specifically from the donor orchid (*Spiranthes brevilabris*) and the location (Dunnellon, Levy Co., FL) from which it was originally obtained. According to Stewart et al. (2003), this fungus was acquired from the last known population of *S. brevilabris* that was reduced to just 152 individuals under legal protection in a small (21,600 ha) state park in Florida. The region bordering the park has experienced high population growth and habitat loss during the past 30 years. While artificial boundaries and legal protection do not always guarantee the security of rare plants such as *S. brevilabris*, it is conceivable that *Tulasnella calospora* 266 may never have been isolated had this area not been designated as a nature preserve prior. As such, this study exemplifies why in situ habitat preservation is essential for the conservation of orchids and other life forms even in areas restricted in size.

For the purposes of ecological restoration, i.e., reintroducing laboratory-grown seedlings into the wild, we urge researchers and land managers to use discretion in the widespread use of *Tulasnella calospora* 266 until more information becomes available on the natural range of this particular fungus. Zettler et al. (2001, 2005), for example, opted not to reintroduce laboratory-grown seedlings of the U.S. Federally endangered Hawaiian endemic, *Platanthera* (*Peristylus*) *holochila*, because the fungus originated from Florida—a distance separated by > 7500 km. Although *Tulasnella calospora* is reported to be global in distribution, it apparently constitutes a diverse fungal assemblage in need of further study. The use of modern molecular techniques currently available is expected to resolve this complex in the coming years which should shed more light into the distribution of *Tulasnella calospora* 266 and its nearest relatives. Recently, Unruh et al. (2019), identified 32 orchid mycorrhizal fungi in North America with the internal transcribed spacer 47 region, and used shallow genome sequencing to functionally annotate these isolates, one of which was *Tulasnella calospora* 266 (UAMH 9824), laying the groundwork for more work.

## Conclusions

For ex situ conservation and educational purposes, using *Tulasnella calospora* 266 may have an immediate impact for use in botanical gardens frequented by the general public. The ongoing use of this fungus at the Missouri Botanical Garden has already generated orchids to the blooming stage which are currently on display. For critically rare species propagated in this manner, cross-pollinating flowers by hand between orchids in captivity could potentially generate a new source of seed for conservation purposes, reducing or circumventing the need to collect seed from wild populations.

## Data Availability

All datasets used and/or analyzed during the current study are available from the corresponding author on reasonable request.
